# (1*S*,3*R*,8*R*,10*R*)-2,2-Di­bromo-3,7,7,10-tetra­methyl­tri­cyclo­[6.4.0.0^1,3^]dodecan-9-one

**DOI:** 10.1107/S1600536813030936

**Published:** 2013-11-16

**Authors:** Ahmed Benharref, Noureddine Mazoir, Jean-Claude Daran, Moha Berraho

**Affiliations:** a Laboratoire de Chimie Biomoléculaires, Substances Naturelles et Réactivité, URAC16, Faculté des Sciences, Semlalia, BP 2390 Bd My Abdellah, 40000 Marrakech, Morocco; b Laboratoire de Chimie de Coordination, 205 Route de Narbone, 31077 Toulouse Cedex 04, France

## Abstract

The title compound, C_16_H_24_Br_2_O was synthesized by three steps from β-himachalene (3,5,5,9-tetra­methyl-2,4a,5,6,7,8-hexa­hydro-1*H*-benzo­cyclo­heptene), which was isolated from essential oil of the Atlas cedar (*Cedrus atlantica*). The mol­ecule is built up from a seven-membered ring to which a six- and a three-membered ring are fused. The six-membered ring shows a chair conformation. One C atom in the seven-membered ring and two methyl groups attached to the ring are disordered over two sets of sites, with an occupancy ratio of 0.658 (7):0.342 (7).

## Related literature

For background to the reactivity and biological properties of β-himachalene, see: El Haib *et al.* (2011 ▶); El Jamili *et al.* (2002 ▶). For related structures, see: Benharref *et al.* (2013 ▶); Oukhrib *et al.* (2013 ▶); Ourhriss *et al.* (2013 ▶). For conformational analysis, see: Cremer & Pople (1975 ▶).
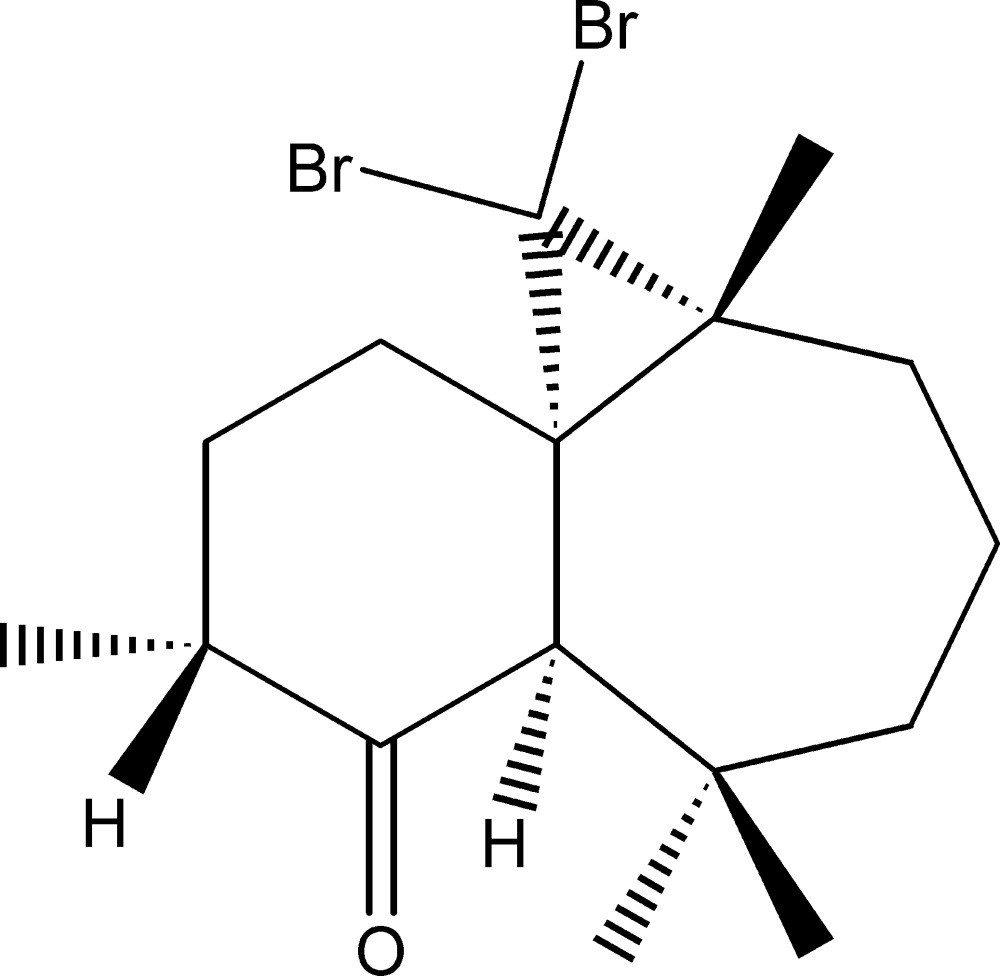


## Experimental

### 

#### Crystal data

C_16_H_24_Br_2_O
*M* *_r_* = 392.17
Monoclinic, 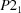

*a* = 6.5975 (2) Å
*b* = 15.2612 (3) Å
*c* = 8.2688 (2) Å
β = 100.045 (3)°
*V* = 819.79 (4) Å^3^
*Z* = 2
Cu *K*α radiation
μ = 6.19 mm^−1^
*T* = 180 K
0.5 × 0.03 × 0.03 mm

#### Data collection

Agilent Xcalibur (Eos, Gemini ultra) diffractometer
Absorption correction: multi-scan (*CrysAlis PRO*; Agilent, 2013 ▶) *T*_min_ = 0.269, *T*_max_ = 1.000
6201 measured reflections
2416 independent reflections
2399 reflections with *I* > 2σ(*I*)
*R*_int_ = 0.021
θ_max_ = 60.5°

#### Refinement

*R*[*F* ^2^ > 2σ(*F* ^2^)] = 0.022
*wR*(*F* ^2^) = 0.057
*S* = 1.07
2416 reflections
206 parameters
13 restraints
H-atom parameters constrained
Δρ_max_ = 0.28 e Å^−3^
Δρ_min_ = −0.46 e Å^−3^
Absolute structure: Flack & Bernardinelli (2000 ▶), 1127 Friedel pairs
Absolute structure parameter: 0.01 (2)

### 

Data collection: *CrysAlis PRO* (Agilent, 2013 ▶); cell refinement: *CrysAlis PRO*; data reduction: *CrysAlis PRO*; program(s) used to solve structure: *SHELXS97* (Sheldrick, 2008 ▶); program(s) used to refine structure: *SHELXL97* (Sheldrick, 2008 ▶); molecular graphics: *ORTEP-3 for Windows* (Farrugia, 2012 ▶); software used to prepare material for publication: *WinGX* (Farrugia, 2012 ▶).

## Supplementary Material

Crystal structure: contains datablock(s) I, global. DOI: 10.1107/S1600536813030936/bt6944sup1.cif

Structure factors: contains datablock(s) I. DOI: 10.1107/S1600536813030936/bt6944Isup2.hkl

Supplementary material file. DOI: 10.1107/S1600536813030936/bt6944Isup3.cml

Additional supplementary materials:  crystallographic information; 3D view; checkCIF report

## Figures and Tables

**Fig. 1.**
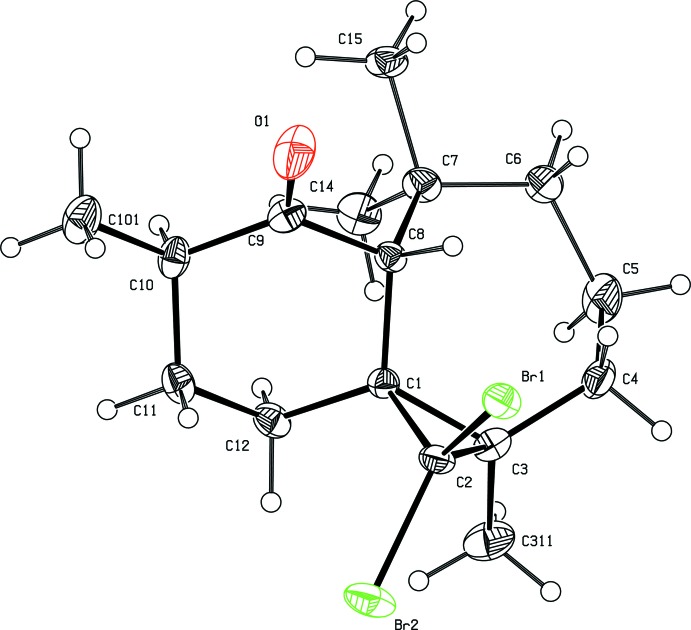
: Molecular structure of the title compound with the atom-labelling scheme. Displacement ellipsoids are drawn at the 30% probability level. H atoms are represented as small spheres of arbitrary radii. The minor occupied sites of the disordered atoms have been omitted for clarity.

## supplementary crystallographic information

### 1. Comment

With the aim exploiting the Moroccan floral inheritance, in particular plants which contain essential oils, we have directed our research endeavours towards the oil of the Atlas Cedar (Cedrus atlantica). The main constituent of this oil is β-himachalene (El Haib *et al.*, 2011). The reactivity of this sesquiterpene has been studied extensively by our team (El Jamili *et al.*, 2002; Benharref *et al.*, 2013; Ourhriss *et al.* (2013), in order to prepare new products having olfactive properieties suitable for the perfume or cosmetics industry. In this work we present the crystal structure of the title compound, (1*S*, 3*R*, 8*R*, 10*R*)-2, 2- dibromo-3,7, 7,10- tetramethyltricyclo[6.4.0.0^1^,^3^]dodecan-9-one. The molecule is built up from two fused seven and six-membered rings and a three-membered ring attached to the seven-membered ring as shown in Fig. 1. The six-membered ring has a chair conformation as indicated by the total puckering amplitude QT = 0.527 (3) Å and spherical polar angle θ = 167.9 (3)° with φ = 99.3 (17)° (Cremer & Pople, 1975). Owing to the presence of Br atoms, the absolute configuration could be successfully confirmed as C1(*S*), C3(*R*), C8(*R*) and C10(*R*).

### 2. Experimental

For the synthesis of the compound (1*S*, 3*R*, 8*R*, 10*R*)-2, 2-dibromo- 3,7,7,10-tetramethyltricyclo [6.4.0.0^1^,^3^]dodecan-9-one, 2 ml of BF_3_—Et_2_O was added dropwise to a 250 ml flask containing a solution of (1*S*,3*R*,8*R*,9S,10*R*)-2,2-dibromo- 9α,10α-epoxy- 3,7,7,10-tetramethyltricyclo-[6.4.0.0^1^,^3^]dodecane (Oukhrib *et al.*,2013) (2 g, 5 mmol) in 100 ml of dichloromethane at 195 K under nitrogen. The reaction mixture was stirred for two hours at a constant temperature of 195 K and was left at ambient temperature for 24 h. Water (60 ml) was a added in order to separate the two phases, and the organic phase was dried and concentrated. The residue obtained was chromatographed on silica- gel eluting with hexane- ethyle acetate (98/2), which allowed the isolation of pure (1*S*, 3*R*, 8*R*, 10*R*)-2, 2- dibromo-3,7, 7,10- tetramethyltricyclo[6.4.0.0^1^,^3^]dodecan-9-one in a Yield 80% (1.56 g, 4 mmol). The title compound was recrystallized from its pentane solution.

### 3. Refinement

All H atoms were fixed geometrically and treated as riding with C—H = 0.96 Å (methyl), 0.97 Å (methylene), 0.98 Å (methine) with *U*_iso_(H) = 1.2U_eq_ (methylene, methine) or *U*_iso_(H) = 1.5*U*_eq_ (methyl). The C6 carbon atom is disordered over two positions inducing a disorder of the two methyl groups C14 and C15 attached to C7. The occupancy factor for these sites was refined.

### Crystal data

**Table:**

C_16_H_24_Br_2_O	*F*(000) = 396
*M**_r_* = 392.17	*D*_x_ = 1.589 Mg m^−^^3^
Monoclinic, *P*2_1_	Cu *K*α radiation, λ = 1.54184 Å
*a* = 6.5975 (2) Å	Cell parameters from 5144 reflections
*b* = 15.2612 (3) Å	θ = 5.4–60.5°
*c* = 8.2688 (2) Å	µ = 6.19 mm^−^^1^
β = 100.045 (3)°	*T* = 180 K
*V* = 819.79 (4) Å^3^	Box, colourless
*Z* = 2	0.5 × 0.03 × 0.03 mm

### Data collection

**Table:**

Agilent Xcalibur (Eos, Gemini ultra) diffractometer	2416 independent reflections
Radiation source: Enhance Ultra (Cu) X-ray Source	2399 reflections with *I* > 2σ(*I*)
Miror monochromator	*R*_int_ = 0.021
Detector resolution: 16.1978 pixels mm^-1^	θ_max_ = 60.5°, θ_min_ = 5.4°
ω scans	*h* = −7→6
Absorption correction: multi-scan (*CrysAlis PRO*; Agilent, 2013)	*k* = −17→17
*T*_min_ = 0.269, *T*_max_ = 1.000	*l* = −9→9
6201 measured reflections	

### Refinement

**Table:**

Refinement on *F*^2^	Secondary atom site location: difference Fourier map
Least-squares matrix: full	Hydrogen site location: inferred from neighbouring sites
*R*[*F*^2^ > 2σ(*F*^2^)] = 0.022	H-atom parameters constrained
*wR*(*F*^2^) = 0.057	*w* = 1/[σ^2^(*F*_o_^2^) + (0.0402*P*)^2^ + 0.1798*P*] where *P* = (*F*_o_^2^ + 2*F*_c_^2^)/3
*S* = 1.07	(Δ/σ)_max_ = 0.001
2416 reflections	Δρ_max_ = 0.28 e Å^−^^3^
206 parameters	Δρ_min_ = −0.46 e Å^−^^3^
13 restraints	Absolute structure: Flack & Bernardinelli (2000), 1127 Friedel pairs
Primary atom site location: structure-invariant direct methods	Absolute structure parameter: 0.01 (2)

### Special details

**Table:**

Experimental. 'Empirical absorption correction using spherical harmonics, implemented in SCALE3 ABSPACK scaling algorithm. CrysAlisPro (Agilent Technologies, 2013 )
Geometry. All e.s.d.'s (except the e.s.d. in the dihedral angle between two l.s. planes) are estimated using the full covariance matrix. The cell e.s.d.'s are taken into account individually in the estimation of e.s.d.'s in distances, angles and torsion angles; correlations between e.s.d.'s in cell parameters are only used when they are defined by crystal symmetry. An approximate (isotropic) treatment of cell e.s.d.'s is used for estimating e.s.d.'s involving l.s. planes.
Refinement. Refinement of *F*^2^ against ALL reflections. The weighted *R*-factor *wR* and goodness of fit *S* are based on *F*^2^, conventional *R*-factors *R* are based on *F*, with *F* set to zero for negative *F*^2^. The threshold expression of *F*^2^ > 2σ(*F*^2^) is used only for calculating *R*-factors(gt) *etc*. and is not relevant to the choice of reflections for refinement. *R*-factors based on *F*^2^ are statistically about twice as large as those based on *F*, and *R*- factors based on ALL data will be even larger.

### Fractional atomic coordinates and isotropic or equivalent isotropic displacement parameters (Å^2^)

**Table:**

	*x*	*y*	*z*	*U*_iso_*/*U*_eq_	Occ. (<1)
Br1	0.99187 (4)	0.122716 (16)	0.78691 (3)	0.03410 (11)	
Br2	0.52030 (5)	0.08850 (2)	0.71788 (4)	0.04828 (14)	
C1	0.7175 (4)	0.19420 (18)	1.0114 (3)	0.0248 (5)	
C2	0.7326 (4)	0.1623 (2)	0.8397 (3)	0.0291 (6)	
C3	0.6907 (5)	0.2575 (2)	0.8643 (3)	0.0326 (7)	
C4	0.8580 (6)	0.3239 (2)	0.8543 (4)	0.0410 (8)	
H4A	0.8283	0.3534	0.7461	0.049*	
H4B	0.9909	0.2928	0.8611	0.049*	
C5	0.8797 (7)	0.3936 (2)	0.9896 (4)	0.0503 (9)	
H5A	0.7420	0.4081	1.0143	0.060*	0.658 (7)
H5B	0.9391	0.4476	0.9509	0.060*	0.658 (7)
H5C	1.0260	0.4115	1.0157	0.060*	0.342 (7)
H5D	0.7990	0.4457	0.9457	0.060*	0.342 (7)
C6	1.0237 (8)	0.3596 (3)	1.1520 (6)	0.0392 (15)	0.658 (7)
H6A	1.1545	0.3392	1.1218	0.047*	0.658 (7)
H6B	1.0568	0.4102	1.2269	0.047*	0.658 (7)
C7	0.9428 (5)	0.2883 (2)	1.2449 (4)	0.0328 (7)	
C14	0.7534 (9)	0.3152 (3)	1.3157 (8)	0.0413 (14)	0.658 (7)
H14A	0.7838	0.3687	1.3809	0.062*	0.658 (7)
H14B	0.7166	0.2682	1.3858	0.062*	0.658 (7)
H14C	0.6383	0.3261	1.2258	0.062*	0.658 (7)
C15	1.1166 (8)	0.2667 (3)	1.3978 (6)	0.0365 (13)	0.658 (7)
H15A	1.2357	0.2410	1.3593	0.055*	0.658 (7)
H15B	1.0630	0.2250	1.4701	0.055*	0.658 (7)
H15C	1.1584	0.3207	1.4585	0.055*	0.658 (7)
C14A	1.1692 (14)	0.3197 (7)	1.2660 (14)	0.045 (3)	0.342 (7)
H14D	1.1823	0.3768	1.3210	0.067*	0.342 (7)
H14E	1.2096	0.3250	1.1579	0.067*	0.342 (7)
H14F	1.2588	0.2772	1.3325	0.067*	0.342 (7)
C15A	0.866 (2)	0.2882 (7)	1.4049 (13)	0.044 (3)	0.342 (7)
H15D	0.9478	0.2471	1.4808	0.065*	0.342 (7)
H15E	0.7213	0.2702	1.3864	0.065*	0.342 (7)
H15F	0.8787	0.3472	1.4521	0.065*	0.342 (7)
C6A	0.8140 (16)	0.3676 (5)	1.1417 (10)	0.039 (3)	0.342 (7)
H6A1	0.6676	0.3499	1.1154	0.047*	0.342 (7)
H6A2	0.8219	0.4196	1.2140	0.047*	0.342 (7)
C8	0.9178 (4)	0.20156 (19)	1.1383 (3)	0.0246 (6)	
H8	1.0321	0.2026	1.0731	0.030*	
C9	0.9510 (5)	0.1190 (2)	1.2426 (4)	0.0317 (6)	
C10	0.7707 (5)	0.07818 (19)	1.3066 (4)	0.0325 (6)	
H10	0.7333	0.1186	1.3919	0.039*	
C11	0.5854 (5)	0.0733 (2)	1.1673 (4)	0.0360 (7)	
H11A	0.6147	0.0305	1.0844	0.043*	
H11B	0.4645	0.0519	1.2117	0.043*	
C12	0.5341 (4)	0.1611 (2)	1.0847 (4)	0.0324 (6)	
H12A	0.5006	0.2040	1.1660	0.039*	
H12B	0.4123	0.1550	0.9967	0.039*	
C101	0.8255 (6)	−0.0096 (2)	1.3886 (4)	0.0468 (9)	
H10A	0.8667	−0.0504	1.3089	0.070*	
H10B	0.7056	−0.0331	1.4293	0.070*	
H10C	0.9396	−0.0019	1.4808	0.070*	
C311	0.4754 (6)	0.2933 (3)	0.7987 (5)	0.0508 (10)	
H31A	0.4663	0.3099	0.6832	0.076*	
H31B	0.4496	0.3448	0.8628	0.076*	
H31C	0.3726	0.2481	0.8081	0.076*	
O1	1.1188 (4)	0.08460 (19)	1.2698 (4)	0.0608 (8)	

### Atomic displacement parameters (Å^2^)

**Table:**

	*U*^11^	*U*^22^	*U*^33^	*U*^12^	*U*^13^	*U*^23^
Br1	0.03127 (18)	0.04181 (18)	0.03046 (17)	−0.00328 (14)	0.00880 (13)	−0.00787 (14)
Br2	0.0335 (2)	0.0641 (3)	0.0454 (2)	−0.01004 (16)	0.00180 (17)	−0.02435 (18)
C1	0.0237 (14)	0.0255 (12)	0.0249 (13)	0.0003 (12)	0.0029 (11)	0.0001 (11)
C2	0.0238 (13)	0.0362 (14)	0.0263 (12)	−0.0054 (13)	0.0016 (11)	−0.0060 (12)
C3	0.0347 (16)	0.0360 (15)	0.0258 (13)	0.0046 (13)	0.0011 (12)	0.0022 (12)
C4	0.060 (2)	0.0321 (16)	0.0289 (15)	−0.0066 (15)	0.0024 (15)	0.0089 (13)
C5	0.077 (3)	0.0221 (15)	0.052 (2)	−0.0032 (16)	0.0115 (19)	0.0029 (14)
C6	0.057 (4)	0.023 (2)	0.035 (3)	−0.011 (2)	0.000 (2)	−0.005 (2)
C7	0.0376 (16)	0.0305 (16)	0.0301 (14)	−0.0034 (13)	0.0056 (14)	−0.0104 (13)
C14	0.053 (3)	0.029 (2)	0.042 (3)	0.001 (3)	0.010 (3)	−0.015 (2)
C15	0.044 (3)	0.029 (2)	0.032 (2)	−0.003 (2)	−0.006 (2)	−0.0076 (19)
C14A	0.038 (6)	0.045 (5)	0.052 (6)	−0.010 (5)	0.008 (5)	−0.019 (5)
C15A	0.067 (8)	0.030 (5)	0.037 (6)	−0.008 (5)	0.020 (6)	−0.015 (4)
C6A	0.052 (7)	0.024 (4)	0.045 (5)	0.002 (4)	0.016 (4)	−0.004 (4)
C8	0.0238 (14)	0.0258 (13)	0.0247 (13)	−0.0026 (12)	0.0053 (11)	−0.0044 (11)
C9	0.0354 (16)	0.0320 (14)	0.0267 (12)	0.0054 (16)	0.0027 (12)	−0.0014 (15)
C10	0.0455 (18)	0.0254 (13)	0.0297 (14)	0.0012 (14)	0.0152 (13)	0.0005 (11)
C11	0.0316 (15)	0.0377 (17)	0.0417 (16)	−0.0064 (14)	0.0149 (13)	−0.0003 (14)
C12	0.0224 (14)	0.0378 (14)	0.0379 (14)	−0.0011 (13)	0.0081 (12)	−0.0046 (14)
C101	0.070 (3)	0.0292 (16)	0.0422 (18)	0.0018 (16)	0.0138 (17)	0.0041 (14)
C311	0.048 (2)	0.050 (2)	0.049 (2)	0.0172 (16)	−0.0070 (18)	0.0059 (15)
O1	0.0390 (13)	0.0750 (18)	0.0715 (17)	0.0250 (14)	0.0177 (12)	0.0388 (15)

### Geometric parameters (Å, º)

**Table:**

Br1—C2	1.934 (3)	C15—H15A	0.9800
Br2—C2	1.938 (3)	C15—H15B	0.9800
C1—C2	1.521 (4)	C15—H15C	0.9800
C1—C12	1.530 (4)	C14A—H14D	0.9800
C1—C3	1.539 (4)	C14A—H14E	0.9800
C1—C8	1.542 (4)	C14A—H14F	0.9800
C2—C3	1.499 (4)	C15A—H15D	0.9800
C3—C4	1.512 (5)	C15A—H15E	0.9800
C3—C311	1.530 (4)	C15A—H15F	0.9800
C4—C5	1.533 (5)	C6A—H6A1	0.9900
C4—H4A	0.9900	C6A—H6A2	0.9900
C4—H4B	0.9900	C8—C9	1.521 (4)
C5—C6A	1.455 (9)	C8—H8	1.0000
C5—C6	1.590 (6)	C9—O1	1.210 (4)
C5—H5A	0.9900	C9—C10	1.517 (4)
C5—H5B	0.9900	C10—C101	1.516 (4)
C5—H5C	0.9900	C10—C11	1.528 (4)
C5—H5D	0.9900	C10—H10	1.0000
C6—C7	1.484 (6)	C11—C12	1.515 (5)
C6—H6A	0.9900	C11—H11A	0.9900
C6—H6B	0.9900	C11—H11B	0.9900
C7—C15A	1.496 (9)	C12—H12A	0.9900
C7—C14	1.525 (6)	C12—H12B	0.9900
C7—C14A	1.549 (9)	C101—H10A	0.9800
C7—C8	1.583 (4)	C101—H10B	0.9800
C7—C15	1.586 (5)	C101—H10C	0.9800
C7—C6A	1.632 (8)	C311—H31A	0.9800
C14—H14A	0.9800	C311—H31B	0.9800
C14—H14B	0.9800	C311—H31C	0.9800
C14—H14C	0.9800		
			
C2—C1—C12	116.7 (2)	C14—C7—C6A	67.3 (4)
C2—C1—C3	58.67 (19)	C14A—C7—C6A	103.5 (6)
C12—C1—C3	122.0 (2)	C8—C7—C6A	109.6 (4)
C2—C1—C8	118.1 (2)	C15—C7—C6A	144.0 (4)
C12—C1—C8	113.4 (2)	C7—C14—H14A	109.5
C3—C1—C8	117.3 (2)	C7—C14—H14B	109.5
C3—C2—C1	61.27 (19)	C7—C14—H14C	109.5
C3—C2—Br1	121.7 (2)	C7—C15—H15A	109.5
C1—C2—Br1	121.02 (19)	C7—C15—H15B	109.5
C3—C2—Br2	120.0 (2)	C7—C15—H15C	109.5
C1—C2—Br2	120.8 (2)	C7—C14A—H14D	109.5
Br1—C2—Br2	106.78 (14)	C7—C14A—H14E	109.5
C2—C3—C4	119.2 (3)	H14D—C14A—H14E	109.5
C2—C3—C311	118.7 (3)	C7—C14A—H14F	109.5
C4—C3—C311	112.5 (3)	H14D—C14A—H14F	109.5
C2—C3—C1	60.06 (19)	H14E—C14A—H14F	109.5
C4—C3—C1	118.7 (2)	C7—C15A—H15D	109.5
C311—C3—C1	118.4 (3)	C7—C15A—H15E	109.5
C3—C4—C5	113.7 (3)	H15D—C15A—H15E	109.5
C3—C4—H4A	108.8	C7—C15A—H15F	109.5
C5—C4—H4A	108.8	H15D—C15A—H15F	109.5
C3—C4—H4B	108.8	H15E—C15A—H15F	109.5
C5—C4—H4B	108.8	C5—C6A—C7	116.6 (6)
H4A—C4—H4B	107.7	C5—C6A—H6A1	108.1
C6A—C5—C4	116.0 (4)	C7—C6A—H6A1	108.1
C6A—C5—C6	53.5 (5)	C5—C6A—H6A2	108.1
C4—C5—C6	110.9 (3)	C7—C6A—H6A2	108.1
C6A—C5—H5A	57.4	H6A1—C6A—H6A2	107.3
C4—C5—H5A	109.5	C9—C8—C1	110.3 (2)
C6—C5—H5A	109.5	C9—C8—C7	112.7 (2)
C6A—C5—H5B	134.6	C1—C8—C7	115.7 (2)
C4—C5—H5B	109.5	C9—C8—H8	105.8
C6—C5—H5B	109.5	C1—C8—H8	105.8
H5A—C5—H5B	108.1	C7—C8—H8	105.8
C6A—C5—H5C	108.3	O1—C9—C10	120.3 (3)
C4—C5—H5C	108.3	O1—C9—C8	120.1 (3)
C6—C5—H5C	59.4	C10—C9—C8	119.6 (2)
H5A—C5—H5C	142.0	C101—C10—C9	112.3 (3)
H5B—C5—H5C	54.1	C101—C10—C11	113.0 (3)
C6A—C5—H5D	108.3	C9—C10—C11	109.3 (2)
C4—C5—H5D	108.3	C101—C10—H10	107.3
C6—C5—H5D	140.8	C9—C10—H10	107.3
H5A—C5—H5D	56.1	C11—C10—H10	107.3
H5B—C5—H5D	55.3	C12—C11—C10	112.5 (3)
H5C—C5—H5D	107.4	C12—C11—H11A	109.1
C7—C6—C5	117.4 (4)	C10—C11—H11A	109.1
C7—C6—H6A	107.9	C12—C11—H11B	109.1
C5—C6—H6A	107.9	C10—C11—H11B	109.1
C7—C6—H6B	107.9	H11A—C11—H11B	107.8
C5—C6—H6B	107.9	C11—C12—C1	109.9 (2)
H6A—C6—H6B	107.2	C11—C12—H12A	109.7
C6—C7—C15A	131.7 (5)	C1—C12—H12A	109.7
C6—C7—C14	113.1 (4)	C11—C12—H12B	109.7
C15A—C7—C14	40.1 (5)	C1—C12—H12B	109.7
C6—C7—C14A	53.5 (5)	H12A—C12—H12B	108.2
C15A—C7—C14A	111.7 (7)	C10—C101—H10A	109.5
C14—C7—C14A	135.3 (5)	C10—C101—H10B	109.5
C6—C7—C8	109.7 (3)	H10A—C101—H10B	109.5
C15A—C7—C8	118.2 (4)	C10—C101—H10C	109.5
C14—C7—C8	115.3 (3)	H10A—C101—H10C	109.5
C14A—C7—C8	109.0 (4)	H10B—C101—H10C	109.5
C6—C7—C15	106.8 (3)	C3—C311—H31A	109.5
C15A—C7—C15	66.7 (6)	C3—C311—H31B	109.5
C14—C7—C15	106.1 (4)	H31A—C311—H31B	109.5
C14A—C7—C15	55.0 (5)	C3—C311—H31C	109.5
C8—C7—C15	105.1 (3)	H31A—C311—H31C	109.5
C6—C7—C6A	52.2 (4)	H31B—C311—H31C	109.5
C15A—C7—C6A	103.7 (6)		
